# B regulatory cells and monocyte subpopulations in patients with chronic graft-vs-host disease

**DOI:** 10.3325/cmj.2021.62.154

**Published:** 2021-04

**Authors:** Antonija Babić, Lejla Kurić, Ana Zelić, Kerep, Lana Desnica, Antonela Lelas, Milan Milošević, Ranka Serventi-Seiwerth, Nadira Duraković, Zinaida Peric, Marinka Mravak, Stipetić, Ervina Bilic, Romana Čeović, Marko Barešić, Tamara Vukić, Dina Ljubas Kelečić, Sanja Mazić, Ines Bojanić, Ana Hećimović, Ernest Bilic, Renata Zadro, Radovan Vrhovac, Steven Z. Pavletic, Drago Batinić, Dražen Pulanić

**Affiliations:** 1 Department of Laboratory Diagnostics, University Hospital Centre Zagreb, Zagreb, Croatia; 2 Department of Internal Medicine, Division of Hematology, University Hospital Centre Zagreb, Zagreb, Croatia; 3 Department of Environmental Health, Occupational and Sports Medicine, Andrija Štampar School of Public Health, University of Zagreb School of Medicine, Zagreb, Croatia; 4 Department of Internal Medicine, Division of Hematology, University Hospital Center Zagreb and University of Zagreb School of Medicine, Zagreb, Croatia; 5 University Hospital Center Zagreb and University of Zagreb School of Dental Medicine, Zagreb, Croatia; 6 Department of Neurology, University Hospital Center Zagreb and University of Zagreb School of Medicine, Zagreb, Croatia; 7 Department of Dermatology, University Hospital Center Zagreb, University of Zagreb School of Medicine, Zagreb, Croatia; 8 Department of Internal Medicine, Division of Clinical Immunology and Rheumatology University Hospital Center Zagreb, University of Zagreb School of Medicine, Zagreb, Croatia; 9 Department of Rehabilitation and Orthopaedic Aids, University Hospital Center Zagreb, Zagreb, Croatia; 10 Department of Internal Medicine, Section for Clinical Nutrition, University Hospital Center Zagreb, Zagreb, Croatia; 11 Department of Transfusion Medicine and Transplantation Biology, University Hospital Center Zagreb, Zagreb, Croatia; 12 Department of Pulmonology, University Hospital Center Zagreb, Zagreb, Croatia; 13 Department of Pediatrics, University Hospital Center Zagreb and University of Zagreb School of Medicine, Zagreb, Croatia; 14 Department of Laboratory Diagnostics, University Hospital Center Zagreb and University of Zagreb Faculty of Pharmacy and Biochemistry, Zagreb, Croatia; 15 National Cancer Institute, Center for Cancer Research, National Institutes of Health, Bethesda, MD, USA; 16 Department of Laboratory Diagnostics, University Hospital Center Zagreb and University of Zagreb School of Medicine, Zagreb, Croatia

## Abstract

**Aim:**

To assess the correlations of B regulatory cells (Bregs) and monocyte subsets in peripheral blood with the National Institutes of Health (NIH)-consensus-defined clinical manifestations of chronic graft-vs-host disease (cGvHD), in an attempt to establish their role as cellular biomarkers.

**Methods:**

This multidisciplinary prospective study enrolled adult cGVHD patients treated in the University Hospital Center Zagreb and University of Zagreb School of Medicine. Immunophenotypic subpopulations of CD24^high^CD38^high^ Bregs (CD27-, CD27+, and total) and monocyte (classical, intermediate, and non-classical) counts were correlated with demographic, transplant, and cGVHD-related data. Bivariate correlation analysis was performed to evaluate the correlations between Bregs and monocytes subsets and cGVHD organ involvement, as well as cGVHD severity and immunosuppression intensity.

**Results:**

Twenty-two adult patients (54.5% female) with cGVHD were enrolled. The median (range) age was 44.5 years (24-65). All patients were transplanted for hematologic malignancies and 40.9% had severe NIH cGVHD global score. The median time from cGVHD diagnosis to the analysis was 16.6 months (0-176). The organ most frequently affected with cGVHD were the eyes (68.2%), skin (45.5%), lungs (45.5%), and liver (40.9%). Lower total and CD27-Bregs counts were correlated with worse cGVHD severity, higher immunosuppression intensity, and lung cGVHD, in terms of cell count, but also with skin cGVHD, in terms of percentages. Patients with liver and joint/fascia cGVHD had a lower percentage of non-classical monocytes and patients with more severe global NIH score had a higher classical monocytes count.

**Conclusion:**

Different organs affected by cGVHD are differently associated with different subpopulations of Bregs and monocytes.

Allogeneic hematopoietic cell transplantation (alloHCT) is a curative treatment option for an array of severe malignant diseases and non-malignant conditions. Chronic graft-vs-host disease (cGVHD) is a major late complication of alloHCT, influencing immune reconstitution and affecting different organs and tissues (1). cGVHD poses a significant morbidity and mortality burden on long-term survivors, a portion of whom require prolonged systemic immunosuppression (2). Since cGVHD is a complex multisystemic allo- and autoimmune disease, different clinical cGVHD presentations might be underlied by distinct pathophysiological processes.

Recently, major insights have been gained into the pathophysiology of cGVHD and its clinical manifestations (3,4). However, research of specific immunological events and their association to specific phenotypical manifestations is still lacking. The National Institutes of Health (NIH) cGVHD Diagnosis and Staging criteria provided much-needed guidelines for clinicians and researchers, facilitating cGVHD drug approvals and novel therapeutic approaches (5). The proposed three-phase pathophysiological model consists of an initial inflammatory phase, a dysregulated immunity phase, and a tissue fibrosis phase (4). The initial tissue injury of hematopoietic stem-cell recipient caused either by the chemotherapeutic regimen, acute GVHD, or infection, produces a number of antigens, which are processed by components of innate and adaptive immune system. The cascade of immunologic events induces the differentiation of pathogenic Th17 cells. Chronic inflammation occurring in cGVHD is consequent to the inability of regulatory mechanisms to control donor-derived effector immune mechanisms. Pathological fibrosis (excessive accumulation of extracellular matrix) is the underlying pathophysiological mechanism for some of the characteristic cGVHD clinical features, such as superficial and deep skin sclerosis, but other organs can also be involved. Many attempts have been made at finding cGVHD biomarkers, whether molecular or cellular, in peripheral blood to improve early diagnosis, prognostication, or therapy monitoring (6,7). However, due to insufficient understanding of the disease pathophysiology and complexity of clinical manifestation, developing reliable biomarkers for clinical use in cGVHD remains an active research aim.

B cells play an important role in cGVHD pathogenesis (8), and different B cell subsets have already been associated with clinical cGVHD manifestations (9,10). Regulatory B cells (Bregs) secrete IL-10 and have immunosuppressive activity, but they do not share a specific immunophenotype, as different subsets of B lymphocytes are capable to differentiate into Bregs (11). The CD24^high^CD38^high^ B cells subset has been associated with immunosuppressive capacity and regulatory functions in autoimmune diseases (12,13).

Monocytes are mononuclear phagocytes that originate in the bone marrow and have a short life span in the circulation before they migrate to the tissue and differentiate into macrophages and dendritic cells (14). Three different subtypes of monocytes have been recognized according to the expression of surface markers CD14 (co-receptor for lipopolysaccharide) and CD16 (FCγIII receptor) (15): non-classical, intermediate, and classical monocytes. Relative percentages of these three subtypes have been correlated with the activity of various cardiovascular, infectious, and autoimmune diseases (16), but rarely in cGVHD.

This pilot study assessed the subpopulations of peripheral blood regulatory CD24^high^CD38^high^ B-lymphocytes (Bregs: CD27^-^, CD27^+^, total Bregs) and monocytes (classical: CD14^2+^CD16^-^, intermediate: CD14^2+^CD16^+^, and non-classical: CD14^+^CD16^2+^) in patients with different clinical manifestations and severity of cGVHD.

## MATERIALS AND METHODS

### Patients

This multidisciplinary prospective study enrolled adult patients with cGVHD who received alloHCT in the University Hospital Center (UHC) and School of Medicine, University of Zagreb, Croatia from June 2017 to January 2018. The patients were extensively clinically assessed by different specialists with expertise in the field of cGVHD, and laboratory data were collected. An experienced hematologist assessed cGVHD organ and global severity according to the 2005 NIH consensus cGVHD diagnosis and staging criteria (5,17). The NIH cGVHD staging criteria score organs based on their functional impairment. Each organ is assigned a score of 0-3 (0 – no involvement, 3 – the worst involvement). The organs/organ systems scored were the skin, eyes, mouth, lungs, joint and fascia, liver, genitalia (for women), and gastrointestinal tract. The intensity of systemic immunosuppression was scored as mild (single-agent prednisone up to 0.5 mg/kg/day), moderate (single-agent prednisone >0.5 mg/kg/day and/or any single agent/modality), or high (two or more agents/modalities ± prednisone >0.5 mg/kg/day) (18). Clinician's impression of disease activity (“active” or “inactive” cGVHD) and clinician's therapeutic intent in terms of cGVHD management at the time of assessment were noted. The disease was defined as “active” if the clinician decided to increase systemic therapy due to a worsening disease, to substitute systemic therapy due to lack of response, or withdraw systemic therapy due to lack of response. The disease was defined as “non-active” if the practitioner decided to decrease systemic therapy because cGVHD was improving or not to change the systemic therapy because cGVHD was stable. Patients who had not been receiving immunosuppressive therapy at evaluation or did not meet any of the mentioned criteria were categorized as “other” (excluded from the analysis of activity) (6,19). The clinician’s 10-point assessment of cGVHD severity is a part of Form A for assessing cGVHD response to therapy (19). The study was approved by the Ethics Committees of UHC Zagreb and University of Zagreb School of Medicine, and patients were enrolled after signing an approved informed consent form.

### Sample collection and laboratory assessment

**Figure 1:**
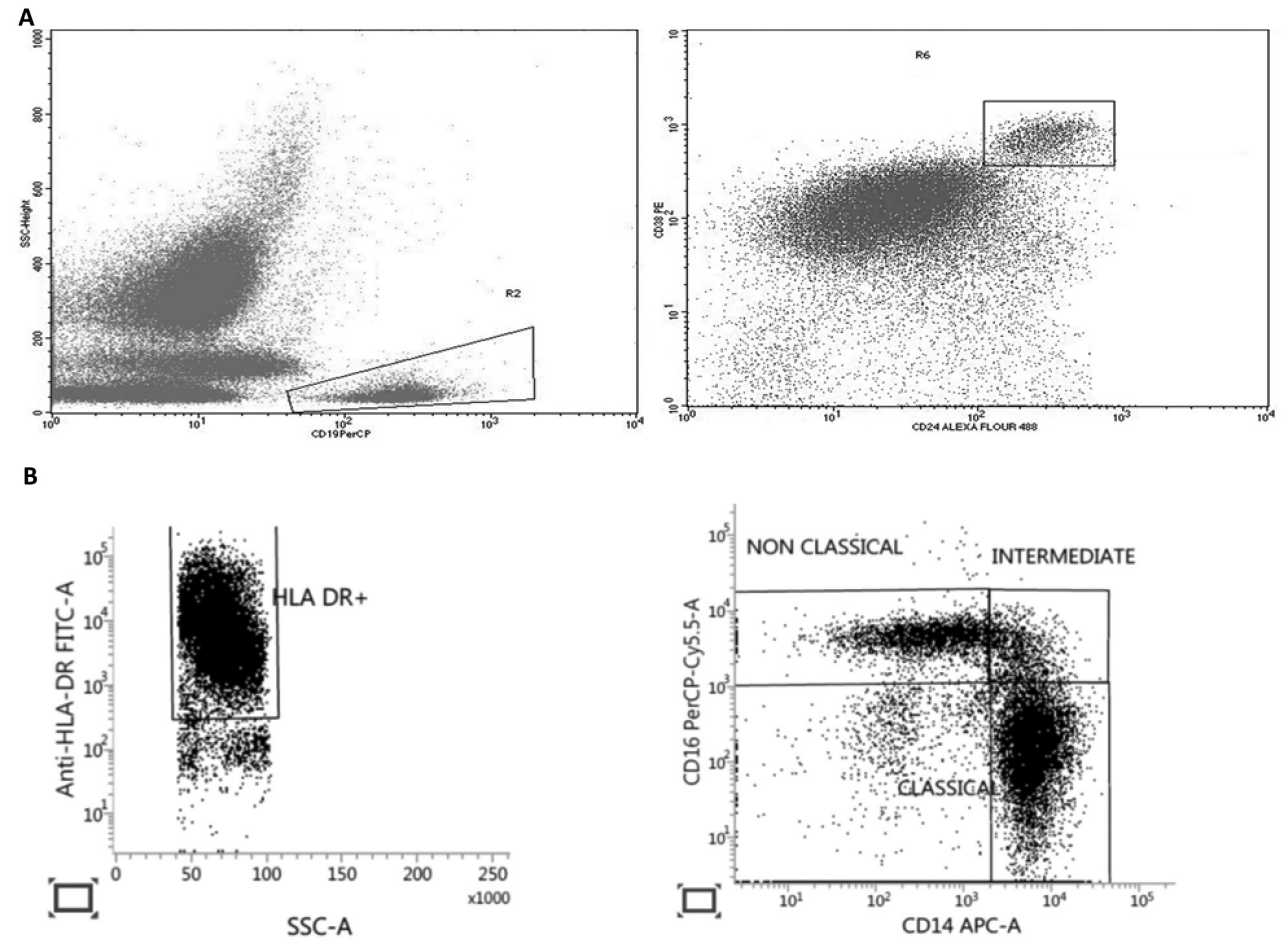
(**A**) Regulatory B cells were gated from CD19 positive cells as CD24++CD38++ cells. (**B**) Monocyte subpopulations were gated from HLA-DR positive cells according to CD14 and CD16 expression as non-classical (CD14+CD16++), intermediate (CD14++CD16+), and classical monocytes (CD14++CD16-). Abbreviations: HLA-DR– human leukocyte antigen-DR; FITC-A – fluorescein isothiocyanate; SSC-A – side scatter; APC-A –allophycocyanin.

Whole blood was collected in K2EDTA blood collection tubes. Blood was divided into two BD-Falcon 5 mL tubes, one for monocyte subsets and one for B lymphocytes. One hundred microliters of blood per tube were incubated for 15 minutes with fluorochrome-conjugated antibodies according to the manufacturer's recommendations, and then erythrocytes were lysed with BD FACS Lysing solution (BD Biosciences, San Jose, CA, USA). The antibodies used for B-lymphocyte characterization were anti-CD24-AlexaFlour488, anti-CD38-PE, anti-CD19-PerCP-Cy5.5, and anti-CD27-APC, and those used for monocyte subsets characterization were anti-HLA-DR-FITC, anti-CX3CR1-PE, anti-CD16-PerCPCy5.5, anti-CD14-APC, anti-CD192-PECy7, and anti-CD183-BV421. All monoclonal antibodies were purchased from BioLegend (San Diego, CA, USA), except anti-HLA-DR-FITC (Dako, Santa Clara, CA, USA) and anti-CD27-APC (eBioscience, San Diego, CA, USA). B-lymphocytes were processed on BD FACS Calibur and analyzed with BD CellQuest software. The cells for monocyte subsets were processed on BD FASCLyric (BD Biosciences) and analyzed using BD FACSuite software. For B-cell analysis, B-cells were first gated according to CD19 expression followed by the identification of CD27^-^ and CD27^+^ B-cell subsets, which were further analyzed according to CD24 and CD38 expression. Two phenotypically distinct B-cell subpopulations with reported regulatory activity were identified as CD27^-^CD24^high^CD38^high^ and CD27^+^CD24^high^CD38^high^ cells (12) (Figure 1A). In monocyte analysis, debris and doublets were first excluded, followed by gating the monocytes according to forward and side scatter (FSC/SSC) parameters. From that, only HLA-DR^+^ cells were gated as monocytes and then analyzed according to CD14 (co-receptor for lipopolysaccharide) and CD16a (Fcγ receptor IIIa) expression (Figure 1B), as well as according to the expression of chemokine receptors CX3CR1 (fractalkine receptor) and CCR2 (CD192). Monocyte subsets were defined as classical monocytes (CD14^2+^CD16^-^) with CX3CR1^low^CCR2^high^, intermediate monocytes (CD14^2+^CD16^+^) with CX3CR1^high^CCR2^mid^, and non-classical monocytes (CD14^+^CD16^2+^) with CX3CR1^high^CCR2^low^ (20). The absolute numbers of monocytes and B lymphocytes were calculated according to white blood count (DxH 800 Beckmann Coulter Inc. Brea, CA, USA).

### Statistical analysis

Descriptive univariate data analyses (number [frequencies], median and range) were used to summarize the patients’ characteristics and laboratory parameters. Bivariate correlation analysis with Spearman's rank correlation test was performed to assess the correlations between Bregs and monocytes subsets with demographic, transplant, NIH cGVHD organ scores, and other cGVHD-related data, as well as other laboratory data. The correlations with moderate or strong Spearman’s rank correlation coefficient>│0.4│ and with statistical significance (*P* < 0.05) were presented. The analysis was performed with IBM SPSS Statistics for Windows, version 25 (IBM Corp., Armonk, NY, USA).

## RESULTS

### Patient population characteristics

The study enrolled 22 adult patients with cGVHD (54.5% female), with the median age of 44.5 years (range 24-65). All were transplanted for hematologic malignancies. The median time from cGVHD diagnosis to the analysis was 16.6 months (0-176). At the moment of analysis, 59.1% were receiving systemic immunosuppression, and the majority had severe (40.9%) or moderate (45.5%) NIH cGVHD global severity score. The patients' characteristics are presented in Table 1. All patients underwent extensive laboratory assessments at enrollment (Table 2).

**Table 1:** Patients' characteristics*

Characteristics	N (%)
Sex	
female	12 (54.5)
male	10 (45.5)
Age at entry, median (range)	44.5 (24-65)
Months from alloHCT to enrollment to the study, median (range)	29.0 (2.9-194.5)
Months from alloHCT to cGVHD, median (range)	9.2 (1.8-28.8)
Underlying malignant disease	
ALL/AML/MDS	13 (59.1)
CML/IMF/myeloproliferative disorders	8 (36.4)
CLL	1 (4.5)
Myeloablative conditioning	
yes	10 (45.5)
no	12 (54.5)
Donor	
unrelated	14 (63.6)
related	8 (36.4)
Cell source	
PBSC	18 (81.8)
BM	4 (18.2)
Previous acute GVHD	
no	9 (40.9)
yes	13 (59.1)
cGVHD onset	
de novo	9 (40.9)
progressive	8 (36.4)
quiscent	5 (22.7)
Clinician's impression of activity	
inactive	14 (63.6)
active	8 (36.4)
NIH cGVHD organ involvement	
eyes	15 (68.2)
lung	10 (45.5)
skin	10 (45.5)
liver	9 (40.9)
mouth	8 (36.4)
joint/fascia	5 (22.7)
genital tract (female only)	4 (33.3)
Global NIH	
mild	3 (13.6)
moderate	10 (45.5)
severe	9 (40.9)
0-10 cGVHD severity	
0	2 (9.1)
1	3 (13.6)
3	1 (4.5)
4	2 (9.1)
5	5 (22.7)
6	1 (4.5)
7	5 (22.7)
8	2 (9.1)
10	1 (4.5)
Lines of prior systemic therapy for cGVHD treatment (number) – including current	
0	5 (27.8)
1	9 (50.0)
2	2 (11.1)
3	2 (11.1)
not known	4 (18.2)
Current intensity of immunosupression	
none	9 (40.9)
mild	0 (0)
moderate	10 (45.5)
high	3 (13.6)

*Abbreviations: alloHCT – allogeneic hematopoietic stem cell transplantation; cGVHD – chronic graft vs host disease; ALL – acute lymphoblastic leukemia; AML – acute myelogenous leukemia; MDS – myelodysplastic syndrome; CML – chronic myelogenous leukemia; IMF – idiopathic myelofibrosis; CLL – chronic lymphocytic leukemia; PBSC – peripheral blood stem cell; BM – bone marrow; NIH – National Institutes of Health.

**Table 2:** Descriptive analysis of laboratory parameters

	Minimum	Median	Maximum	Number of patients
**Leukocytes ×10^9^/L**	2.3	7.9	16.2	22
**Lymphocytes, %**	3.3	36.0	75.0	22
**Lymphocytes/μL**	27.3	268.2	6975.0	22
**Monocytes, %**	2.0	10.2	16.6	22
**Monocytes/μL**	213.9	751.7	1620.0	22
**Non classical, % monocytes**	0.5	4.3	15.1	18
**Non classical, monocytes/μL**	3.3	30.5	123.6	18
**Intermediate, % monocytes**	2.1	7.5	20.1	18
**Intermediate, monocytes/μL**	1.1	5.0	261.4	18
**Classical, % monocytes**	72.8	85.4	97.4	18
**Classical monocytes/μL**	187.8	697.3	1547.7	18
**B lymphocytes, %**	0.5	14.0	44.0	22
**B lymphocytes/μL**	2.0	297.5	1427.0	22
**CD27+ Bregs, % B lymphocytes**	0.0	0.1	0.5	22
**CD27+ Bregs /μL**	0.0	0.3	1.7	22
**CD27- Bregs, % B lymphocytes**	0.0	3.1	29.8	22
**CD27-Bregs/μL**	0.0	11.4	54.1	22
**Total Bregs, % B lymphocytes**	0.0	3.2	30.3	22
**Total Bregs/μL**	0.0	11.6	55.8	22
**Platelets ×10^9^ /L**	94.0	213.5	451.0	22
**Erythrocyte sedimentation (mm/h)**	2.0	30.0	80.0	21
**ALT (U/L)**	11.0	33.5	135.0	22
**CRP (mg/L)**	0.2	3.7	57.7	20
**IgG (g/L)**	4.3	10.8	26.5	19

*Abbreviations: Bregs – B regulatory cells (immunophenotypically defined); ALT – alanine aminotransferase; CRP – C-reactive protein; IgG – Immunoglobulin G.

### The correlations of B-regulatory subpopulations with cGVHD characteristics

**Figure 2:**
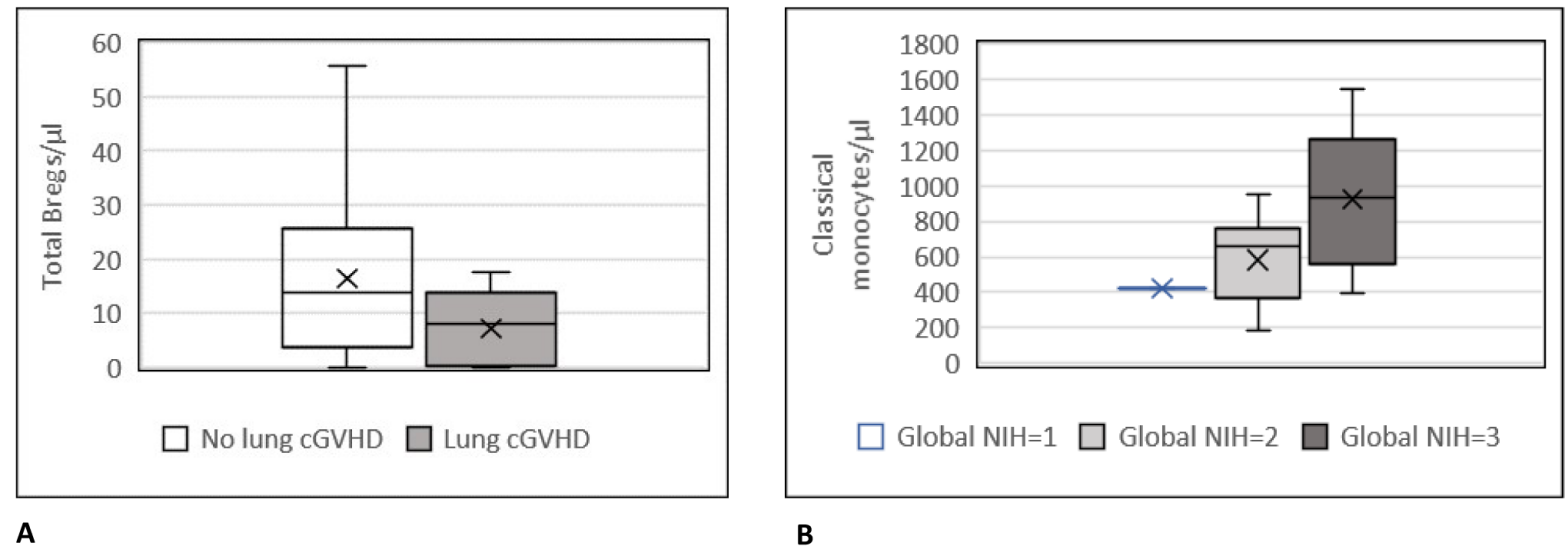
(**A**) Total regulatory B cell count in patients according to their chronic graft vs host disease lung involvement. (**B**) Total classical monocytes count in patients according to their global National Institutes of Health score.

Immunophenotypically defined Bregs subpopulations were analyzed in all patients. As expected, the patients who were receiving more intensive immunosuppression at the time of evaluation had lower total B lymphocytes (*P* = 0.042), as well as total Bregs (*P* = 0.043) and both CD27- (*P* = 0.039) and CD27+ (*P* = 0.021) Bregs count in the peripheral blood. However, lower total Bregs (*P* = 0.026), but not total B lymphocyte count (*P* = 0.715), was correlated with a more severe cGVHD assessed on a 10-point scale (clinicians´ impression of cGVHD severity) (21). Patients with lung cGVHD (Figure 2A) had lower total (*P* = 0.015), CD27^-^ (*P* = 0.021), and CD27^+^ (*P* = 0.037) Bregs cell count. A higher Karnofsky score was correlated with a higher total (*P* = 0.007), CD27^-^ (*P* = 0.008), and CD27^+^ (*P* = 0.019) Bregs count. The liver, eyes, mouth, and joint/fascia cGVHD did not correlate with B lymphocyte subpopulations (Table 3).

**Table 3:** Correlations between B lymphocyte subpopulations and cGVHD-related data (N = 22)

Non-parametric correlation coefficients (Spearman's rho)		B lymphocytes, %	B lymphocytes/μL	CD27+ Bregs, % B lymphocytes	CD27+ Bregs/μL	CD27- Bregs, % B lymphocytes	CD27-Bregs/μL	Total Bregs, % B lymphocytes	Total Bregs/μL
**Lines of prior systemic therapy for cGVHD**	r_S_	-0.306	-0.308	-0.337	-0.538	-0.241	-0.531	-0.257	-0.531
	P	0.216	0.213	0.171	0.021	0.335	0.023	0.303	0.023
**Intensity of immuno-suppression**	r_S_	-0.451	-0.438	-0.124	-0.489	-0.031	-0.442	-0.051	-0.434
	P	0.035	0.042	0.584	0.021	0.889	0.039	0.821	0.043
**Clinician's impression of activity**	r_S_	-0.433	-0.462	0.007	-0.351	0.067	-0.313	0.06	-0.313
	P	0.044	0.031	0.974	0.110	0.767	0.156	0.792	0.156
**Karnofsky score (%)**	r_S_	-0.223	0.047	0.582	0.496	0.615	0.549	0.626	0.557
	P	0.319	0.835	0.005	0.019	0.002	0.008	0.002	0.007
**Lung cGVHD**	r_S_	0.141	-0.05	-0.516	-0.446	-0.442	-0.488	-0.468	-0.51
	P	0.531	0.826	0.014	0.037	0.040	0.021	0.028	0.015
**Eyes cGVHD**	r_S_	-0.436	-0.251	0.219	-0.009	0.329	-0.019	0.313	0.006
	P	0.043	0.259	0.328	0.967	0.135	0.934	0.155	0.98
**Skin cGVHD**	r_S_	0.076	0.295	-0.357	-0.292	-0.458	-0.231	-0.465	-0.245
	P	0.738	0.182	0.103	0.187	0.032	0.302	0.029	0.272
**0-10 cGVHD severity**	r_S_	0.099	-0.082	-0.372	-0.364	-0.347	-0.463	-0.363	-0.473
	P	0.662	0.715	0.089	0.096	0.114	0.030	0.097	0.026

*Abbreviations: cGVHD – chronic graft vs host disease; Bregs – B regulatory cells (immunophenotypically defined).

### The correlations of monocyte subpopulations with cGVHD characteristics

Monocyte subpopulations were not analyzed in 4 out of 22 patients due to technical issues. Patients with joint/fascia cGVHD (*P* = 0.007) and mouth cGVHD (*P* = 0.043) had a lower percentage of non-classical monocytes, as well as patients with liver cGVHD (*P* = 0.043), who also had a lower percentage of intermediate (*P* = 0.007) and a higher percentage of classical monocytes (*P* = 0.004). On the other hand, patients with genital tract cGVHD had a higher percentage of non-classical monocytes (*P* = 0.027). Patients with a more severe global NIH score (*P* = 0.040) had a higher classical monocytes count (Figure 2B) and those with a higher Karnofsky score (*P* = 0.020) had a higher percentage of non-classical monocytes. Lung, eyes, and skin cGVHD and the intensity of immunosuppression did not correlate with monocyte subpopulations (Table 4).

**Table 4:** Correlations between monocyte subpopulations and cGVHD-related data* (N = 18†)

Non-parametric correlation coefficients (Spearman's rho)		Monocytes, %	Monocytes/μL	Non classical, %	Non classical/μL	Intermediate, %	Intermediate/μL	Classical, %	Classical /μL
**Karnofsky score (%)**	r_S_	0.145	-0.035	0.542	0.409	0.193	0.121	-0.27	-0.311
	P	0.518	0.878	0.02	0.092	0.442	0.633	0.278	0.209
**Genital cGVHD**	r_S_	-0.288	-0.64	0.725	0.207	0.207	0.104	-0.518	-0.414
	P	0.391	0.034	0.027	0.593	0.593	0.791	0.154	0.268
**Joint/fascia cGVHD**	r_S_	0.135	0.382	-0.614	-0.379	-0.376	-0.073	0.446	0.466
	P	0.549	0.080	0.007	0.121	0.124	0.772	0.064	0.052
**Liver cGVHD**	r_S_	-0.002	0.159	-0.482	-0.287	-0.612	-0.158	0.649	0.325
	P	0.992	0.479	0.043	0.247	0.007	0.532	0.004	0.189
**Mouth cGVHD**	r_S_	0.144	0.441	-0.158	0.037	0.028	0.371	0.083	0.482
	P	0.524	0.040	0.532	0.884	0.913	0.13	0.742	0.043
**Global NIH cGVHD**	r_S_	0.094	0.139	-0.189	0.004	0.082	0.361	0.177	0.487
	P	0.679	0.538	0.452	0.989	0.746	0.142	0.481	0.040

*Abbreviations: cGVHD – chronic graft vs host disease.

†Four participants were not analyzed for monocyte subpopulations due to technical issues.

Two major primary hematological disease groups (acute leukemia/MDS vs CML/IMF/myeloproliferative disorders) did not significantly differ in monocyte subpopulations and B regulatory cells count (data not shown).

## DISCUSSION

In our study, patients with more severe cGVHD, as assessed by the clinician using the 0-10 cGVHD symptom severity scale (21), had significantly lower Bregs count and patients with better Karnofsky performance status had significantly higher Bregs count. B-cells activate the immune system by presenting antigens, secreting cytokines, and producing antibodies, and also exhibit immunosuppressive activity by IL-10-dependent and IL-10-independent mechanisms (22). Bregs are known as the subsets of B cells with immunosuppressive activity and are able to inhibit the innate and adaptive immune responses (23,24). Bregs in humans were first described by Blair et al, who identified them in peripheral blood within the CD19+CD24^high^CD38^high^ subset corresponding to immature transitional B-cells (12). Another human Breg subpopulation has been described within the activated/memory CD19+CD24^hi^CD27+ B-cell subset (25), and as an IgM+ Breg subpopulation within the CD27+CD24^hi^CD38-/low memory subset (26). The role of B cells in cGVHD is corroborated by the evidence of a breakdown in peripheral B-cell tolerance and altered immune regulation in patients after alloHCT (27), as well as reduced Bregs frequencies in patients with cGVHD compared with allogeneic transplant recipients with no history of cGVHD and healthy donors (26). B-lymphocytes are being recognized as important factors in cGVHD pathogenesis, potential biomarkers (27,28), and promising therapeutic targets (29). For example, the first and currently the only drug approved to treat cGVHD is ibrutinib, an inhibitor of Bruton’s tyrosine kinase, an important enzyme in B cells development (30). In addition, a potential protective effect of Bregs could pave the way for new therapeutic possibilities in the treatment of cGVHD (31).

Our preliminary results also suggest that specific organ involvement correlates with different monocyte subsets. Monocytes are a heterogeneous and functionally highly plastic cell group with an important role in the initiation of inflammation as well as its resolution and healing (20). The majority of peripheral blood monocytes, about 80%, express CD14++CD16- immunophenotype and are termed “classical” monocytes (32). Their primary role is phagocytosis and initiation of inflammation (33). The remaining 20% express CD16 and are divided into intermediate CD14++CD16+ (inflammatory subtype, contributing to local and systemic inflammation) (34) and non-classical CD14+CD16++ subtype, the so-called patrolling monocytes. This subtype has a role in anti-inflammatory and wound healing processes (35). Joint/fascia involvement in our study was significantly associated with lower percentages of patrolling monocyte subsets, as well as liver involvement. Since non-classical monocytes have the highest CX3CR1 expression among monocyte subsets, their decrease in the blood would suggest the monocytes infiltration to the disease sites via CX3CR1/fractalkine pathway, in line with Namba et al (36). Liver involvement was also associated with lower percentages of inflammatory, but a higher percentage of classical monocyte subset. Classical monocytes were also found to be associated with a higher NIH global severity score and mouth cGVHD. In contrast, Konuma et al (37), who investigated circulating monocyte subsets in cGVHD patients, found no difference in the numbers and the proportions of circulating monocyte subsets. However, their study revealed that altered expressions of activation markers and chemokine and scavenger receptors on each monocyte subset were significantly associated with specific organ involvement of cGVHD. Hirayama et al have found that an absolute number of intermediate monocytes, as well as high CD29 expression, correlates with cGVHD activity (38).

Chronic graft-vs-host disease is a complex immunological disorder, challenging to diagnose and treat, with a therapeutic response that is hard to sustain. Clinical manifestations overlap with other conditions occurring in the post-transplant period, whether it is an infection, medication side effects, or consequences of previous chemotherapeutic regimens and the underlying disease itself (19). Many organ systems can be involved, leaving patients with different levels of functional impairment and diminished quality of life (39-42). The strength of this study is the prospective clinical data collection and adherence to rigorous assessments using standardized cGVHD NIH diagnostic criteria in an interdisciplinary setting of experienced subspecialists.

The results of this study add to the current understanding of cGVHD biology (3), as relates to the possible role of Bregs and monocyte subpopulations. These results indicate a possible biological difference in some of the cGVHD clinical features. Circulating monocytes were not affected by the immunosuppression level, which, along with the fact that they may be considered reporters of immunological processes, makes monocytes an excellent candidate for future cGVHD biomarker research. Although this pilot study analyzed a relatively small number of patients, it observed a strong association between cGVHD pathophysiology and clinical manifestations, warranting future research in larger cohorts with longitudinal sample collection and with a control population of alloHCT recipients without cGVHD. Bregs and monocyte subpopulations in patients after alloHCT need to be longitudinally followed up to assess the importance of changes in these cell populations in cGVHD prediction and its severity, as well as in response to different treatment modifications.

Our results add to the recently growing body of knowledge of cGVHD immunology in humans, facilitating the future development of better-targeted and personalized therapeutic options.
